# A Novel Model for Acute Peripheral Nerve Injury in the Horse and Evaluation of the Effect of Mesenchymal Stromal Cells Applied *In Situ* on Nerve Regeneration: A Preliminary Study

**DOI:** 10.3389/fvets.2016.00080

**Published:** 2016-09-15

**Authors:** Claudia Cruz Villagrán, Jim Schumacher, Robert Donnell, Madhu S. Dhar

**Affiliations:** ^1^Department of Large Animal Clinical Sciences, College of Veterinary Medicine, University of Tennessee, Knoxville, TN, USA; ^2^Department of Biomedical and Diagnostic Sciences, College of Veterinary Medicine, University of Tennessee, Knoxville, TN, USA

**Keywords:** horse, nerve regeneration, peripheral nerve, stem cells, mesenchymal stromal cells, neuropathies

## Abstract

Transplantation of mesenchymal stromal cells (MSCs) to sites of experimentally created nerve injury in laboratory animals has shown promising results in restoring nerve function. This approach for nerve regeneration has not been reported in horses. In this study, we first evaluated the *in vitro* ability of equine bone marrow-derived MSCs (EBM-MSCs) to trans-differentiate into Schwann-like cells and subsequently tested the MSCs *in vivo* for their potential to regenerate a transected nerve after implantation. The EBM-MSCs from three equine donors were differentiated into SCLs for 7 days, *in vitro*, in the presence of specialized differentiation medium and evaluated for morphological characteristics, by using confocal microscopy, and for protein characteristics, by using selected Schwann cell markers (GFAP and S100b). The EBM-MSCs were then implanted into the fascia surrounding the *ramus communicans* of one fore limb of three healthy horses after a portion of this nerve was excised. The excised portion of the nerve was examined histologically at the time of transection, and stumps of the nerve were examined histologically at day 45 after transplantation. The EBM-MSCs from all donors demonstrated morphological and protein characteristics of those of Schwann cells 7 days after differentiation. Nerves implanted with EBM-MSCs after nerve transection did not show evidence of nerve regeneration at day 45. Examination of peripheral nerves collected 45 days after injury and stem cell treatment revealed no histological differences between nerves treated with MSCs and those treated with isotonic saline solution (controls). The optimal delivery of MSCs and the model suitable to study the efficacy of MSCs in nerve regeneration should be investigated.

## Introduction

Horses suffer injury to peripheral nerves from trauma, metabolic disease, toxins, genetic disorders, and degenerative and infectious diseases (1–3). Sequelae to nerve injury in horses frequently include poor performance, disability, or even death, which, in turn, result in profound financial and emotional burden.

Schwann cells form the myelin sheath surrounding axons in the peripheral nervous system. This sheath is regularly segmented by the nodes of Ranvier, which help in transmitting the nerve impulse in a saltatory and, thus, extremely fast manner (4). Peripheral nerves are able to regenerate after injury due to the secretion of cytokines and neurotrophic factors emanating from the damaged cells (especially from Schwann cells) and to phagocytosis of cellular debris by local macrophages (5–7).

Residual nerve function depends on the magnitude and chronicity of damage to the nerve (8). The prognosis for recovery of a nerve is poorest when the nerve is transected, and its fibers and surrounding fascia (connective tissue) have been completely disrupted. This magnitude of damage is known as neurotmesis in Seddon’s classification of peripheral nerve injury (9, 10). Surgical techniques for repairing nerves involve apposing the stumps of the nerve with sutures or inserting a graft to bridge the gap between the stumps (5, 6). Some of the most commonly used materials for fabricating grafts or scaffolds consist of vein, artery, nerve, silicone, collagen, laminin, gels made of platelet-rich plasma (PRP), and synthetic polymers (11, 12). Regardless of the technique of tissue engineering used, clinical results are often disappointing (10, 13).

Results of research using laboratory animals indicate that using mesenchymal stromal cells (MSCs) might be an alternative approach for repairing nervous tissue, including injury to the spinal cord and peripheral nerves (14–16). The neuroprotective effects of MSCs have been widely described and involve anti-inflammatory, immunomodulatory, angiogenic, and nurturing mechanisms (17–20). Furthermore, MSCs from bone marrow or adipose tissue are able to differentiate into Schwann-like cells (SLCs) after being chemically induced under specific conditions (16, 21, 22). SLCs transplanted into experimentally created nervous lesions of laboratory animals have enhanced repair of axons and myelin and improved sensory and motor functions, perhaps as a consequence of the secretion of specific neurotrophic factors that promote nerve repair (15, 16, 20).

There are no reports describing outcomes of horses treated for peripheral nerve injury with equine bone marrow-derived MSCs (EBM-MSCs) or any report that describes the ability of EBM-MSCs to differentiate into myelinated SLCs. These studies are necessary before EBM-MSCs can be used in veterinary medicine to enhance regeneration of damaged peripheral nerves. We have recently reported that EBM-MSCs are able to display morphological and protein characteristics of neural progenitors (23). In this preliminary study, we evaluated the ability of EBM-MSCs to differentiate into SLCs after chemical induction, *in vitro*. Additionally, we describe a new model of peripheral nerve injury in the horse and discuss the outcome after implanting undifferentiated EBM-MSCs into an experimentally created nerve lesion in three horses.

## Materials and Methods

### Isolation, Expansion, and Characterization of EBM-MSCs

Bone marrow-derived MSCs, obtained from the sternum, previously characterized and cryopreserved from three equine donors, were assessed for their stem cell properties, as described (19). Briefly, all cells were confirmed to be mesenchymal stromal/stem cells based on their colony-forming unit assays, MTS viability and proliferation assays, and the adipogenic, chondrogenic, and osteogenic differentiation patterns, as described in previous studies (24). Low-passage MSCs (P1 to P4) isolated from three equine donors were used in all *in vitro* experiments described below.

### Trans-Differentiation of EBM-MSCs into Schwann-Like Cells

Low-passage equine MSCs were seeded at a density of 8 to 10 × 10^6^ cells onto 100 mm Primaria™ nitrogen-coated tissue culture dishes (Becton Dickinson Labware, Bedford, MA, USA). Cells were maintained in regular growth medium containing Dulbecco’s modified Eagle medium/Ham’s F-12 (DMEM-F12) (Cellgro™, Manassas, VA, USA), 10% fetal bovine serum (FBS), and 1% penicillin/streptomycin, at 37°C and 5% CO_2_, for 48 h to allow attachment. Neural differentiation was induced using a previously described method (16). To induce neural differentiation, the growth medium was removed, and cells were pre-incubated with medium containing DMEM-F12, 20% FBS, and 1 mM β-mercaptoethanol (Sigma-Aldrich^®^, Saint Louis, MO, USA), at 37°C and 5% CO_2_, for 18–24 h. Subsequently, the cells were induced by adding neural medium containing DMEM-F12, 10% FBS, and 35 ng/mL all-*trans*-retinoic acid. The medium was replaced after 3 days with DMEM-F12, 10% FBS, and a mixture of cytokines containing the following ingredients: 5 μM forskolin (Sigma-Aldrich^®^, Saint Louis, MO, USA), 200 ng/mL recombinant human heregulin-β1 (HRG-β1) (Sigma-Aldrich^®^, Saint Louis, MO, USA), 5 ng/mL platelet-derived growth factor (Sigma-Aldrich^®^, Saint Louis, MO, USA), and 10 ng/mL recombinant human basic fibroblast growth factor (Sigma-Aldrich^®^, Saint Louis, MO, USA). The cells were incubated in this medium, which was replaced every 72 h, for 7 days.

For cytoplasmic staining, SLCs and undifferentiated EBM-MSCs were stained with 5 μg of wheat-germ agglutinin (WGA, Alexa Fluor^®^ 488 conjugate) (Life Technologies™, Grand Island, NY, USA) for 10 min, at room temperature. To stain the nucleus, cells were washed and stained with 5 μg of TO-PRO^®^-3 iodide stain (Life Technologies™, Grand Island, NY, USA) for 10 min, at room temperature. The cells were washed and mounted with Slowfade^®^ Gold antifade reagent (Molecular Probes^®^, Grand Island, NY, USA) and photographed through a laser-scanning spectral confocal microscope (Leica TCS SP2) (Leica Microsystems©, Wetzlar, Germany), at 20× and 63× magnification.

Total cell lysates were prepared from SLCs and undifferentiated EBM-MSCs using 200 μL of RIPA buffer (Boston Bioproducts™, Ashland, MA, USA) and sonicated, and supernatants containing total proteins were recovered by centrifugation. Total proteins in each sample were quantitated, and concentrations were determined using BCA assay at 660 nm (Pierce^®^, Thermo Scientific™, Waltham, MA, USA). Equal concentrations (20 μg per lane) of total proteins from SLCs and undifferentiated MSCs were electrophoretically separated in a 12% acrylamide gel and transferred onto nitrocellulose membranes. The membranes were blocked with 5% bovine serum albumin (BSA) and incubated with mouse anti-S-100b (BD Pharmingen™, San Diego, CA, USA) (1:1000), mouse anti-β_3_ tubulin (Santa Cruz Biotechnology™, USA) (1:1000), and mouse anti-GFAP (BD Pharmingen™, San Diego, CA, USA) (1:1000). Horseradish peroxidase (HRP)-conjugated goat anti-mouse IgG (BD Pharmingen™, San Diego, CA, USA) (1:5000) was used as the secondary antibody. Antigens were detected after being exposed to ECL-2 reagent (Pierce^®^, Thermo Scientific™, Waltham, MA, USA). Beta-actin was used as a loading control.

To observe proteins using immunofluorescence, SLCs and undifferentiated EBM-MSCs were fixed with 4% paraformaldehyde, permeabilized with 0.1% Triton X-100 (Sigma-Aldrich^®^, Saint Louis, MO, USA) for 10 min, at room temperature, and blocked with 5% normal serum for 30 min, at room temperature. Cells were washed and incubated overnight, at 4°C, with 5 μg of primary antibodies against S-100b (BD Pharmingen™, San Diego, CA, USA) and GFAP (BD Pharmingen™, San Diego, CA, USA). After being washed with HBSS buffer, cells were incubated with 5 μg Alexa Fluor^®^ 647 donkey anti-mouse IgG (BD Pharmingen™, San Diego, CA, USA), for 20 min, at room temperature, mounted with Slowfade^®^ Gold antifade with DAPI reagent (Molecular Probes^®^, Grand Island, NY, USA), and photographed through a laser-scanning spectral confocal microscope (Leica Microsystems©, Wetzlar, Germany).

### Subjects

Three healthy American Quarter Horse, crossbred mares, 9–13 years old, from the University of Tennessee’s teaching herd were used in the study. All procedures were carried out according to a protocol approved by the Institutional Animal Care and Use Committee.

### Equine Model of Peripheral Nerve Injury

**Figure 1:**
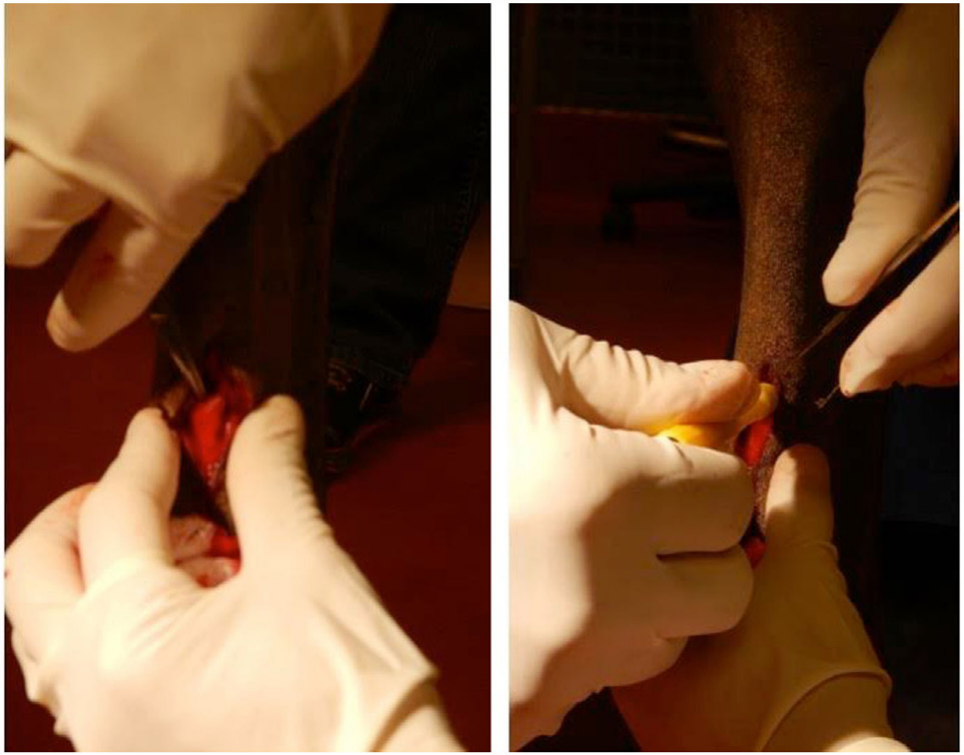
**Equine model of peripheral nerve injury**. Dissection (left) and transection (right) of the *ramus communicans* nerve of a fore limb of a horse. A 6-mm diameter biopsy punch was used to transect the center of the nerve.

Horses were sedated with detomidine hydrochloride (0.01–0.02 mg/kg, IV) and butorphanol tartate (0.01–0.02 mg/kg, IV). The distal palmar aspect of both metacarpi was prepared for aseptic surgery, and 2 mL of 2% mepivacaine hydrochloride was deposited subcutaneously adjacent to the medial palmar nerve and adjacent to lateral palmar nerve proximal to the palpable *ramus communicans* lying palmar to the superficial digital flexor tendon. A scalpel blade was used to create a 15-mm long, cutaneous, longitudinal incision over the *ramus communicans*. Using a 6-mm diameter biopsy punch, the center of this anastomotic nerve, connecting the medial and lateral palmar nerves, was removed and placed in 10% formalin (Figure 1). The same procedure was performed on the contralateral fore limb.

### Treatments and Histological Analysis of Nerve Tissue

**Figure 2:**
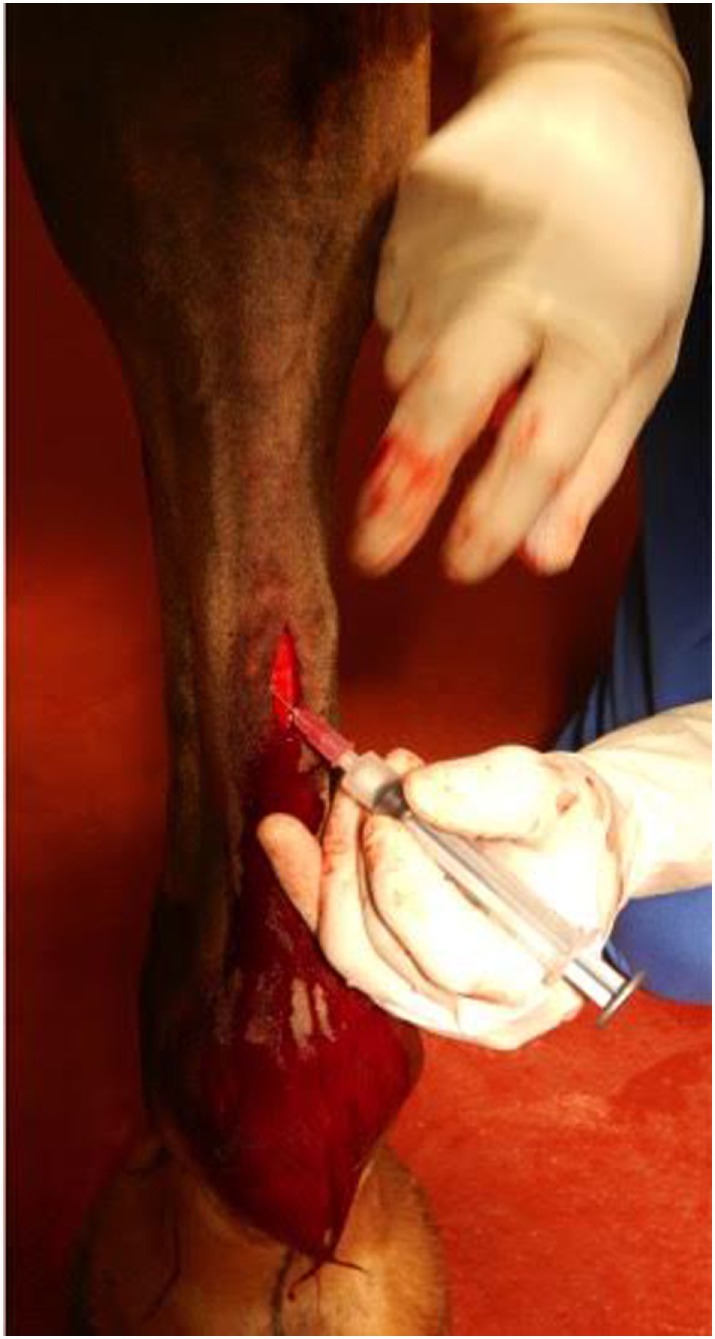
**Transplantation of allogeneic EBM-MSCs into the fascia surrounding the *ramus communicans* of a fore limb of a horse following nerve transection**.

After excising a portion of the *ramus communicans* of one randomly selected fore limb, 10 × 10^6^ allogeneic, undifferentiated MSCs (from a mare, whose MSCs had been previously characterized), suspended in 1 mL of sterile isotonic saline solution were instilled into the fascia surrounding the medial and lateral stumps of the *ramus communicans* (Figure 2). The same volume of sterile isotonic saline solution was injected around the stumps of the contralateral nerve (control). The cutaneous incision was closed with staples, and the distal portion of each fore limb was bandaged. The horses received phenylbutazone at the time of surgery (4.4 mg/kg, PO) and the day after surgery (2.2 mg/kg, PO). The limbs were bandaged until the staples were removed on day 14. Bandages were changed every third day.

The stumps of *ramus communicans* of each fore limb of each horse (*n* = 3) were harvested 45 days post stem cell therapy. To remove the stumps, the horses were sedated with xylazine hydrochloride (0.5 mg/kg, IV) and anesthetized with ketamine hydrochloride (2.2 mg/kg, IV). General anesthesia was maintained with isofluorane vaporized in oxygen and delivered through a semi-closed system into a cuffed endotracheal tube inserted orally into the trachea. The horses were placed in right lateral recumbency. A cutaneous, 2-cm, longitudinal incision was created over the medial and lateral aspects of the flexor tendons of both fore limbs to expose the medial and lateral stumps of the *ramus communicans*. The medial and lateral stumps of the nerve were transected close to the medial or lateral palmar nerve and placed in Carson’s fixative until processed for histological examination. The same procedure was performed on the contralateral fore limb, and the incisions were closed with staples, and the distal portion of each fore limb was bandaged. All horses recovered uneventfully from anesthesia. The limbs were bandaged until the staples were removed on day 14. Bandages were changed every third day.

Five-micrometer thick sections of Carson’s fixed, paraffin-embedded nerve excised from the *ramus communicans* and from the stumps of the *ramus communicans* were examined by light microscopy.

## Results

No horses showed sign of discomfort or became lame after the surgical procedures, and none of the horses exhibited any sign of immunological response after allogenic EBM-MSCs were implanted in the nerve defects.

### Schwann Cell Differentiation of EBM-MSCs *In Vitro*

**Figure 3:**
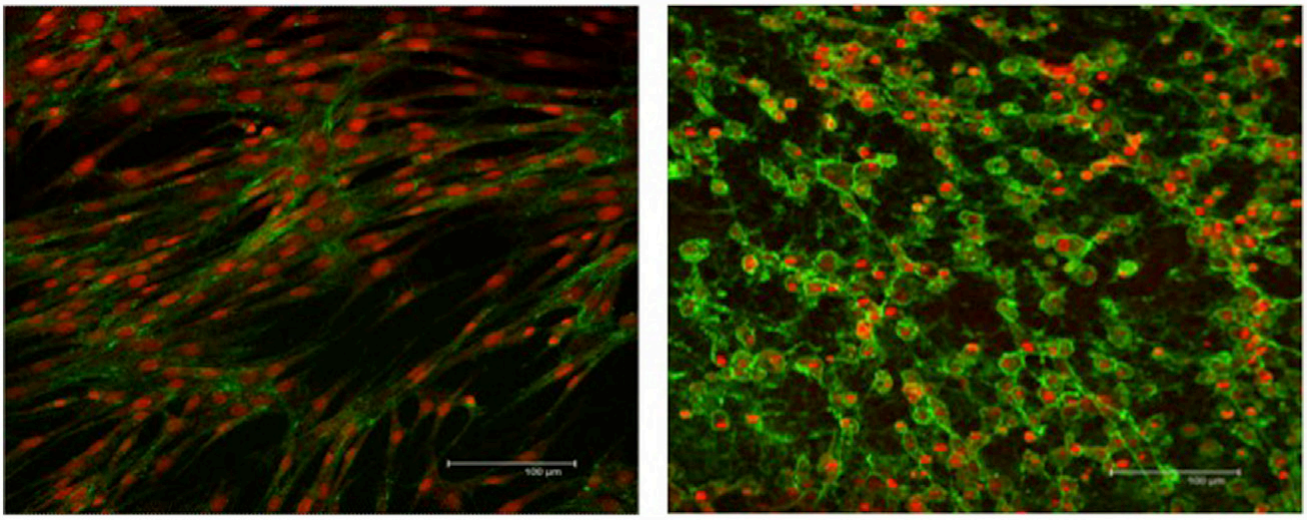
**WGA cytoplasmic and TO-PRO-3-iodide nuclear immunofluorescent stains showing the integrity of EBM-MSCs (left) and SLCs (right)**. Note the typical fibroblast-like morphology of the EBM-MSCs (control), the elongation of the SLCs into a spindle shape, the appearance of one or more cellular processes, and the growth of cells into a “whorl-like” pattern, at day 7. Scale bar = 100 μm.

The integrity of the nucleus and cytoplasm of the cells was examined using fluorescence microscopy. TO-PRO^®^-3 stain and WGA, specific to the cell membrane, were used to demonstrate the nucleus and the cytoplasmic structure of the SLCs. Low-passage EBM-MSCs demonstrated the potential to undergo trans-differentiation after being chemically induced for 7 days. Trans-differentiation was observed subjectively using morphological changes in cells exposed to the differentiation medium relative to the undifferentiated controls. The cells elongated until they displayed spindle-shaped morphology, accompanied by the appearance of one or two cell processes (Figure 3). These cells grew in a “whorl-like” pattern, a phenotype typical of Schwann cells. Cells undergoing differentiation displayed this morphological change at 4 days after chemical induction, and approximately 80% of the cells had acquired this morphologic change by 7 days. The undifferentiated controls maintained the typical fibroblastic appearance of a MSC throughout the 7-day period. Subjectively, no differences were observed in the phenotypic characteristics of SLCs, generated from EBM-MSCs, among equine donors.

**Figure 4:**
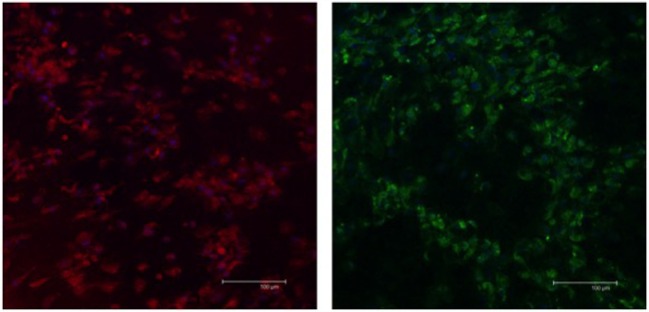
**Expression of Schwann-cell proteins in EBM-MSCs trans-differentiated into Schwann-like cells**. Confocal microscopy shows the expression of S100b (red) and GFAP (green) in Schwann-like cells from a middle-aged horse after 7 days of chemical induction. Scale bar = 100 μm.

**Figure 5:**
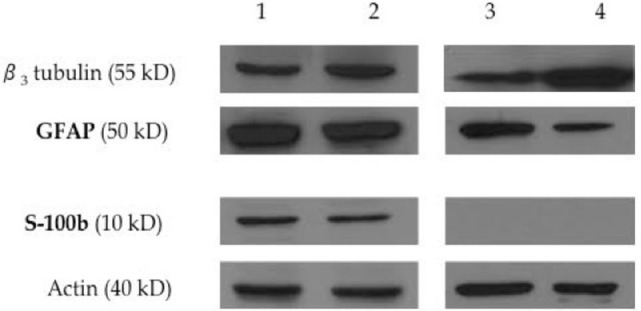
**Expression of neural progenitor and Schwann-cell proteins by immunoblot analysis**. Western blot analysis shows the expression of the neural progenitor proteins β_3_ tubulin and GFAP, and the expression of the Schwann-cell protein S100b on equine Schwann-like cells from middle-aged (lane 1) and young (lane 2) horses. Undifferentiated EBM-MSCs show the expression of the neural progenitor proteins β_3_ tubulin and GFAP on cells from middle-aged (lane 3) and young (lane 4) horses. Beta-actin was used as an internal control. Twenty micrograms of protein was loaded per lane. Note the expression of GFAP on undifferentiated MSCs, suggesting that the plasticity of EBM-MSCs may go beyond mesodermal lineages. S100b could not be detected by immunoblots. Undifferentiated MSCs did not show Schwann-cell protein expression.

The expression of S-100b and GFAP was confirmed by a combination of immunofluorescence (Figure 4) and immunoblot (Figure 5) analyses. The expression profiles were confirmed in SLCs differentiated from the EBM-MSCs of all equine donors. Interestingly, immunoblot analyses showed that the undifferentiated control cells expressed β_3_ and GFAP, but failed to express S-100b. Results suggest that β_3_ and GFAP may be neural progenitor markers, and their expression can be used as an indicator to demonstrate plasticity (i.e., the ability of equine MSCs to differentiate into other cellular lineages beyond that of the mesodermal lineages).

### Peripheral Nerve Regeneration

**Figure 6:**
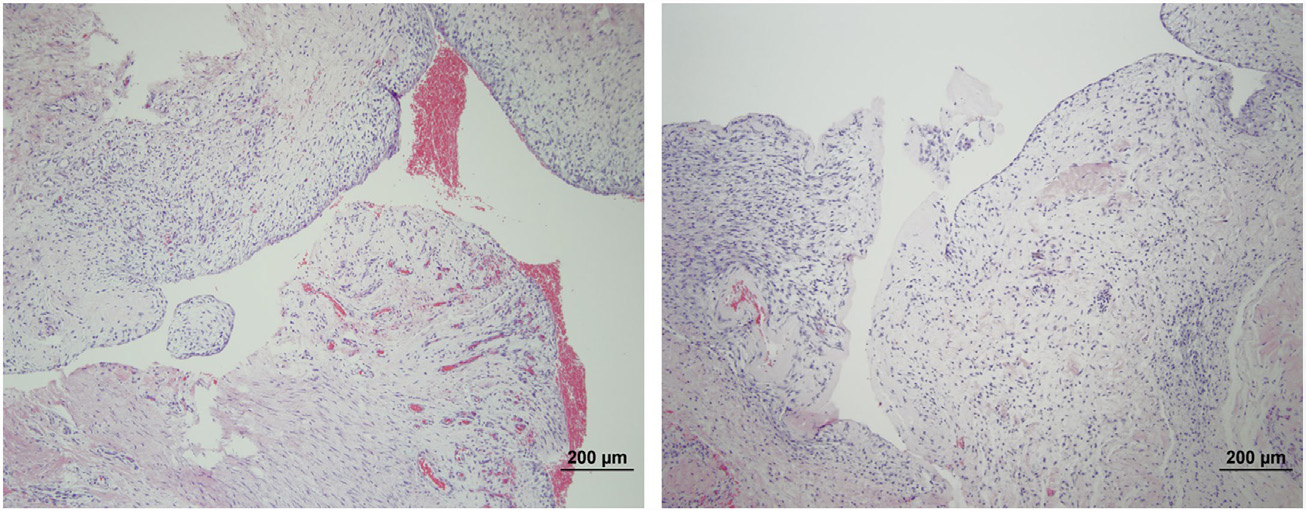
**Formation of seromas at the transection sites**. Hematoxylin and eosin staining showed all nerves treated with saline solution (left) and MSCs (right) had cleft-like spaces rimmed by or partially filled by aggregates of fibrin overlain or infiltrated by fibroblastic cells and macrophages. 100× magnification.

**Figure 7:**
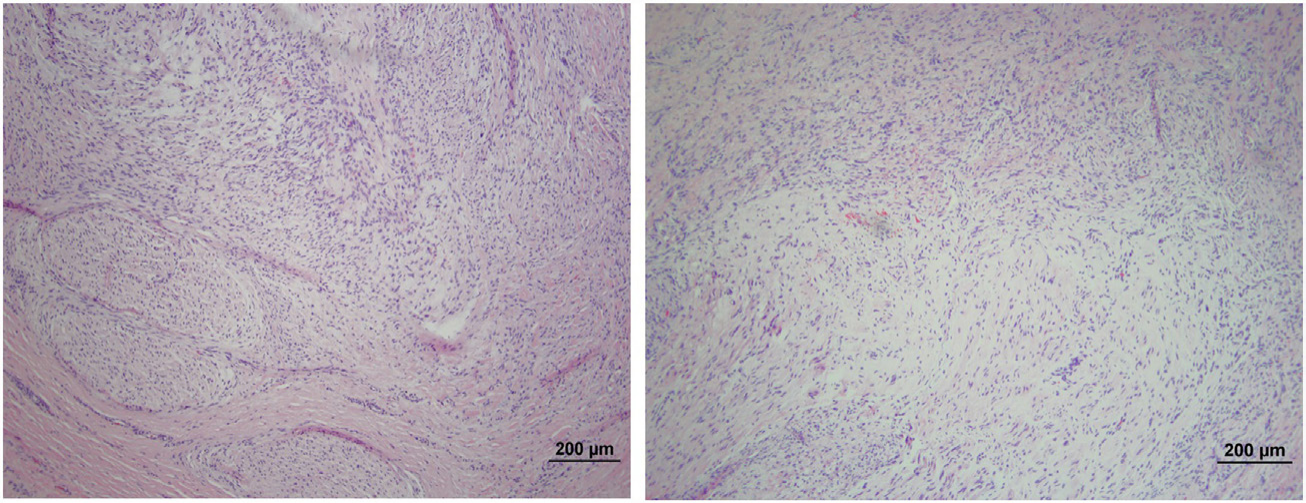
**Formation of post-traumatic neuromas on peripheral nerves 45 days after nerve transection**. Hematoxylin and eosin staining showed that both the isotonic saline solution-treated (left) and the MSCs-treated (right) nerves formed neuromas characterized by haphazard streams, whorls, and fascicles of small vessels, fibroblasts, and Schwann cells. No population of MSCs or primitive neural cells was recognized. 100× magnification.

The microscopic appearance of the nerves in all specimens transected with the punch biopsy on the first surgical procedure was within normal limits. On day 45, all stumps treated with isotonic saline solution and all stumps treated with MSCs had seromas characterized by cleft-like spaces rimmed by, or partially filled by, aggregates of fibrin overlain, bordered, or partially infiltrated by fibroblastic cells and macrophages (Figure 6). The isotonic saline-treated and MSC-treated stumps had formed post-traumatic, transectional neuromas characterized by haphazard streams, whorls, and fascicles of small vessels, fibroblasts, and Schwann cells (Figure 7). No localized or discrete population of MSCs or primitive cells (e.g., neural or Schwann-cell progenitors) was recognized.

## Discussion

Peripheral nerves can be injured by chemical, thermal, or mechanical trauma (25). Direct trauma, metabolic disease, such as hypoparathyroidism, electrolyte imbalance, and equine motor neuron disease, and idiopathic disease, such as damage to the left recurrent laryngeal nerve (recurrent laryngeal neuropathy), appear to be the nerve injuries most commonly reported in the horse. Treating horses for damage to a peripheral nerve, by administering one or more anti-inflammatory drugs and physical rehabilitation, is often unrewarding. Repair of transected nerve fibers of human beings is enhanced by closing the gap by apposing the nerve endings and their surrounding fascia with sutures or by inserting an autograft (11). The use of autografts in horses, however, might not be practical and could result in unwanted sequelae (26).

Techniques investigated to repair damaged nerves using regenerative therapies include transplantation of MSCs, alone or in combination with bioengineered materials, for the purpose of providing proper environmental conditions for survival and proliferation of cells that can aid nerve repair, particularly Schwann cells (20, 27). Based on experimental studies in laboratory animals, the transplantation of MSCs after peripheral nerve injury speeds regain of motor and sensory nerve functions (15, 20, 28, 29). Additionally, studies have demonstrated that MSCs are capable of extra-mesodermal differentiation, including differentiation into cells from neural lineage. Transplantation of MSCs differentiated into SLCs on to injured nerves has revealed promising results on nerve function and morphology (14, 15, 30).

We chemically induced low-passage, bone marrow-derived MSCs from young and middle-aged horses to determine their plasticity into SLCs. Morphological changes, determined by phase-contrast and fluorescence microscopic examinations, became evident by 7 days after chemical induction in all horses. Differentiated cells were elongated, had an oval-shaped cytoplasm, and had formed one or multiple cellular processes. These cells appeared to multiply to form patches, whereas undifferentiated MSCs multiplied to form flat, even layers.

Western blot and immunofluorescence analyses revealed the expression of the Schwann cell markers S-100b and GFAP in SLCs. Undifferentiated MSCs also expressed β_3_ and GFAP, as previously reported by our group (23), but not S-100b. Expression of β_3_ and GFAP suggests that EBM-MSCs are capable of differentiating into cells of extra-mesodermal lineages (31).

The *in vitro* portion of this study relied mainly on morphological analysis and Schwann-cell protein markers to describe the events occurring during differentiation of EBM-MSCs into SLCs. Our results were similar to those experiments performed using bone marrow-derived MSCs from rats and humans, in which cells were able to differentiate and express markers typical of Schwann cells (7, 21, 30, 32).

The *in vivo* portion of this study used a model for acute peripheral nerve injury in the horse, which has not previously been reported. As opposed to other models of peripheral nerve injury, transecting the *ramus communicans* does not cause sensory or functional impairment in the horse. This is an important welfare matter. Additionally, we evaluated the effects on speed and pattern of nerve regeneration after transplantation with allogeneic MSCs. Transplanting MSCs that had differentiated into SLCs was not possible due to poor viability of the differentiated cells (25% viable) after the cells were detached from the tissue culture flasks. A method to optimize collection of equine SLCs is necessary. Furthermore, no histological differences were observed between nerve stumps treated with MSCs and those treated with isotonic saline solution (controls).

For this specific study, we had previously characterized the stemness of the MSCs from all our equine donors. Horses with various diseases presented to our institution have been treated with allogeneic MSCs previously characterized and stored. We have subjectively perceived benefits from transplanting these allogeneic cells into damaged tendons and ligaments. Our study was aimed at differentiating allogeneic MSCs into SLCs for treating horses for nerve injuries because the ultimate aim of our research is to have cells readily available to use for regenerating damaged nerves, because to regenerate nerves, expeditious treatment after injury is critical for success. Allogeneic MSCs can be used as an “off the shelf” product soon after injury, whereas culturing and expanding autogenous MSCs requires several days.

Results were inconclusive using our model for acute peripheral nerve injury in the horse, perhaps because of the low number of horses used and perhaps because of other factors, such as surgical technique, anatomical region of the experimentally injured nerve (the distal aspect of the limb of the horse has less vasculature than other areas in the body), method of cellular delivery, and labeling and tracking of cells. A portion of the *ramus communicans* may, perhaps, be excised with less trauma to adjacent tissue with the horse anesthetized than with the horse standing. With better exposure, the cells could, perhaps, be injected directly into the nerve stumps. Similarly, suturing the incision in the fascia and subcutaneous tissue may prevent the formation of seromas.

To the best of our knowledge, this is the first report describing the morphological features and protein expression changes of EBM-MSCs into SLCs. In this study, we validated the cross-reactivity of rat Schwann cell-specific antibodies with protein samples from horses. Further studies exploring the viability, homing, and functionality of MSCs and SLCs after transplantation into horses with peripheral nerve injuries are warranted. Similarly, a practical and affordable method for delivering these cells is necessary.

## Author Contributions

CCV, JS, and MD designed and conceived the work. CV and MD acquired and analyzed all data. CCV and MD carried out all *in vitro* experiments. CCV and JS carried out all *in vivo* experiments and acquired the nerve tissues. RD analyzed the nerve tissues. CCV, JS, and MD drafted the manuscript, and all authors revised it and approved the last version for publication. All authors agree to be accountable for all aspects of the work ensuring that questions related to the accuracy or integrity of any part of the work are appropriately investigated and resolved.

## Conflict of Interest Statement

This research was conducted in the absence of any commercial or financial relationships that could be construed as a potential conflict of interest.

## Abbreviations

EBM-MSCs, equine bone marrow-derived mesenchymal stromal cells; GFAP, glial fibrillary acidic protein; MSCs, mesenchymal stromal cells; SLCs, Schwann-like cells.
